# Tumor-induced senescent T cells promote the secretion of pro-inflammatory cytokines and angiogenic factors by human monocytes/macrophages through a mechanism that involves Tim-3 and CD40L

**DOI:** 10.1038/cddis.2014.451

**Published:** 2014-11-06

**Authors:** M C Ramello, J Tosello Boari, F P Canale, H A Mena, S Negrotto, B Gastman, A Gruppi, E V Acosta Rodríguez, C L Montes

**Affiliations:** 1 Centro de Investigaciones en Bioquímica Clínica e Inmunología (CIBICI-CONICET), Departamento de Bioquímica Clínica, Facultad de Ciencias Químicas, Universidad Nacional de Córdoba, Haya de la Torre y Medina Allende, Ciudad Universitaria, Córdoba, Argentina; 2 Laboratorio de Trombosis Experimental, Instituto de Medicina Experimental (IMEX), Academia Nacional de Medicina-CONICET, Buenos Aires, Argentina; 3 Cleveland Clinic, Department of Immunology and Plastic Surgery, Head and Neck and Taussig Institutes, Cleveland, OH, USA

## Abstract

Solid tumors are infiltrated by immune cells where macrophages and senescent T cells are highly represented. Within the tumor microenvironment, a cross-talk between the infiltrating cells may occur conditioning the characteristic of the *in situ* immune response. Our previous work showed that tumors induce senescence of T cells, which are powerful suppressors of lympho-proliferation. In this study, we report that Tumor-Induced Senescent (TIS)-T cells may also modulate monocyte activation. To gain insight into this interaction, CD4+ or CD8+TIS-T or control-T cells were co-incubated with autologous monocytes under inflammatory conditions. After co-culture with CD4+ or CD8+TIS-T cells, CD14+ monocytes/macrophages (Mo/Ma) exhibit a higher expression of CD16+ cells and a reduced expression of CD206. These Mo/Ma produce nitric oxide and reactive oxygen species; however, TIS-T cells do not modify phagocyte capacity of Mo/Ma. TIS-T modulated-Mo/Ma show a higher production of pro-inflammatory cytokines (TNF, IL-1*β* and IL-6) and angiogenic factors (MMP-9, VEGF-A and IL-8) and a lower IL-10 and IP-10 secretion than monocytes co-cultured with controls. The mediator(s) present in the supernatant of TIS-T cell/monocyte-macrophage co-cultures promote(s) tubulogenesis and tumor-cell survival. Monocyte-modulation induced by TIS-T cells requires cell-to-cell contact. Although CD4+ shows different behavior from CD8+TIS-T cells, blocking mAbs against T-cell immunoglobulin and mucin protein 3 and CD40 ligand reduce pro-inflammatory cytokines and angiogenic factors production, indicating that these molecules are involved in monocyte/macrophage modulation by TIS-T cells. Our results revealed a novel role for TIS-T cells in human monocyte/macrophage modulation, which may have deleterious consequences for tumor progression. This modulation should be considered to best tailor the immunotherapy against cancer.

Clinical and experimental studies have established that several types of solid tumors are characterized by infiltration of both innate and adaptive immune cells. Indeed, it has been reported that tumors can be infiltrated by different cell populations such as B cells, NK cells, Th1 and Th2 cells, regulatory T cells (Tregs), senescent T cells and macrophages, among others.^1, 2, 3^ Investigating the nature and effector function of these tumor-infiltrating subsets is highly relevant as accumulating evidence indicates that a dynamic cross-talk between tumors and immune system cells can regulate tumor growth and metastasis.^4, 5^

Macrophages constitute a major component of the leukocytes that infiltrate tumors. Tumor-associated macrophages (TAMs) derive almost entirely from circulating monocytes, which acquire distinct phenotypic characteristics and diverse functions according to the tumor microenvironment. Prototypically, two different types of activated macrophages have been recognized: the classically activated (M1) or pro-inflammatory macrophages and the alternatively activated (M2) macrophages. Thus, in response to diverse signals like cytokines or membrane receptor ligation, macrophages undergo M1 or M2 polarization states characterized by particular profiles of cytokine and chemokine production. M1 macrophages express high levels of pro-inflammatory cytokines, major histocompatibility complex (MHC) molecules and inducible nitric oxide (NO) sintetase. By contrast, M2 macrophages downregulate MHC class II and show increased expression of the anti-inflammatory cytokine IL-10 and mannose receptor. In addition, macrophages can also be polarized into a M2-like state, which shares some but not all the signature features of M2 cells.^1^ Macrophages with intermediate or overlapping phenotypes have been observed in many pathological conditions *in vivo*, probably as a result of the effect of diverse signals that occur along different times of the immune response. In fact, many studies emphasize the heterogeneity and plasticity of macrophages and indicate that typical M1 and M2 phenotypes are extremes of a wide spectrum of functional states.^6, 7, 8^

Within the tumor microenvironment, the macrophages interact with or receive signals from different tumor-infiltrating immune cells such as Tregs, myeloid-derived suppressor cells, Th1 and Th2 cells, among others.^1, 9^ This interaction may regulate the profile of macrophage activation and consequently impact on tumor progression. It has been described that the excessive activity of either M1 or M2 subsets may be detrimental to the host by preventing the development of an efficient anti-tumor immune response.^10^ Understanding the cellular interactions that lead to the control of monocyte/macrophage (Mo/Ma) activation is, therefore, of fundamental importance to the field of tumor immunology.

Senescent T cells are reported to be increased during chronic infections and some tumor processes.^11, 12^ In fact, senescent T cells circulate in the peripheral blood of most cancer patients and infiltrate tumors.^2^ Although the hallmark of human senescent T cells is the loss of CD27 and CD28 expression, other features of these cells include shortened telomeres, reduced proliferative capacity and cytokine production as well as suppressor activity.^13, 14^ We previously reported that, after a brief contact with tumor cells, CD4+ as well as CD8+ T lymphocytes from healthy donors acquire a senescent phenotype. These CD4+ and CD8+ tumor-induced senescent (TIS)-T cells are characterized by the loss of CD27 and CD28 expression, lack of proliferative capacity, telomere shortening and increment in the expression of senescence-associated molecules such as p53, p21 and p16. Remarkably, these CD4+ and CD8+ TIS-T populations also show a potent suppressive ability.^15^ We also demonstrated that tumor-induced senescence of T cells is triggered by soluble factors secreted by tumor cells and that this process can be prevented by IL-7.^16^ These data support the hypothesis that, within the tumor microenvironment, tumor-infiltrating T lymphocytes encounter tumor cells that promote their senescence and dysfunction. These TIS-T cells would be able to suppress the lympho-proliferative response and potentially modulate other immune cells. Thus, they may serve as an intercellular cross-talk in the tumor microenvironment and impact on tumor progression.

Although macrophages and TIS-T lymphocytes are both highly represented in tumors, the biological consequences of a TIS-T cell and macrophage interaction have not been studied so far. Here, we demonstrate that monocytes co-cultured with TIS-T cells in inflammatory conditions increase their production of inflammatory cytokines and angiogenic factors. In addition, we determined that Mo/Ma modulation mediated by TIS-T lymphocytes requires cell-to-cell contact and identified T-cell immunoglobulin and mucin protein 3 (Tim-3) and CD40 ligand (CD40L) molecules as mediators of this previously uncharacterized modulatory function of senescent T cells.

## Results

### TIS-T lymphocytes induce classical activation in Mo/Ma

**Figure 1:**
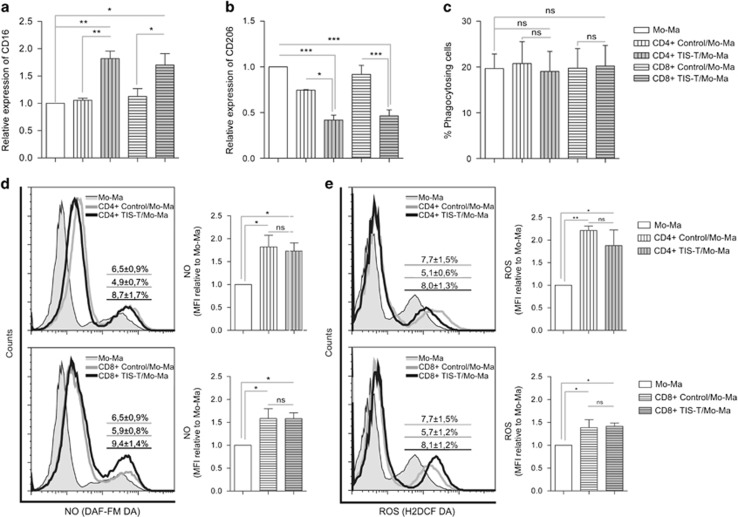
CD4+ and CD8+ TIS-T cells induce classical activation in monocytes/macrophages. Monocytes were cultured alone, with CD4+ or CD8+ control T cells, or with CD4+ or CD8+ TIS-T cells (in 1:1 ratio), in the presence of anti-CD3 mAb (2 *μ*g/ml). The phenotype of Mo/Ma and their ability to produce NO and ROS was assessed after 40 h of co-culture. (**a**) CD16 expression (average MFI±S.E.M.) on Mo/Ma co-cultured with TIS-T cells or control T cells relative to Mo/Ma cultured alone. (**b**) CD206 expression (average MFI±S.E.M.) on Mo/Ma co-cultured with TIS-T cells or control T cells relative to Mo/Ma cultured alone. (**c**) Percentage±S.E.M. of phagocytosing CD14+ cells. *P<0.05, **P<0.01, ***P<0.001, ns, not significant (*P*>0.05); statistical analysis were performed by using one way ANOVA test (*n*=4). Histograms represent (**d**) NO and (**e**) ROS production by Mo/Ma co-cultured with CD4+ (upper graphs) or CD8+ (lower graphs) T cells. Percentages±S.E.M. (*n*=4) of NO- and ROS-producing CD14+ cells are indicated in the histograms. (NO: *P*=0.011, CD4+ TIS-T/Mo-Ma *versus* CD4+ control T/Mo-Ma and *P*=0.014 CD8+ TIS-T/Mo-Ma *versus* CD8+ control T/Mo-Ma. ROS: *p*=0.041 CD4+ TIS-T/Mo-Ma *versus* CD4+ control T/Mo-Ma and *p*=0.006 CD8+ TIS-T/Mo *versus* CD8+ control T/Mo-Ma). Bar graphs represent MFI of NO and ROS-CD14+ producing cells relative to Mo/Ma cultured alone. *P<0.05, **P<0.01, ns, not significant (*P*>0.05); statistical analysis were performed using one-way ANOVA test (*n*=4)

To study whether CD4+ or CD8+ TIS-T lymphocytes can steer the differentiation of human monocytes, CD14+ monocytes were cultured alone or with autologous CD4+ or CD8+ TIS-T lymphocytes or control CD4+ or CD8+ T lymphocytes stimulated with anti-CD3 mAb. After 40 h of culture, phenotype markers were evaluated by flow cytometry on CD14+ cells. We first analyzed the expression of CD16, typically associated with pro-inflammatory or classically activated monocytes. As determined by the higher mean of fluorescence intensity (MFI), we found a significantly increased expression of CD16 on Mo/Ma co-cultured with CD4+ or CD8+ TIS-T cells than on those co-cultured with control counterparts (Figure 1a). These control co-cultures (CCs) exhibited similar expression of CD16 than Mo/Ma cultured alone (Figure 1a). In addition, we observed a reduced expression of CD206, an alternative activation marker, in Mo/Ma cultured with CD4+ or CD8+ TIS-T lymphocytes compared to Mo/Ma cultured alone or with CD4+ or CD8+ control-T lymphocytes (Figure 1b). In contrast, no differences were detected in the expression of HLA-DR and co-stimulatory molecules such as CD86 and B7.H4 (data not shown). To further evaluate the features of the Mo/Ma cultured with TIS-T cells, we studied phagocytic activity, shown to be increased in alternatively activated human macrophages.^1^ In line with their phenotype of classically activated cells, Mo/Ma co-incubated with CD4+ or CD8+ TIS-T lymphocytes did not modify their phagocyte capacity (Figure 1c).

To establish whether the acquisition of a classically activated phenotype is accompanied by the corresponding effector function, we evaluated Mo/Ma ability to produce reactive nitrogen and oxygen species. The percentages of NO and Reactive Oxygen Species (ROS)-producing CD14+ cells, were higher in cultures of Mo/Ma with CD4+ or CD8+ TIS-T lymphocytes compared with those with control CD4+ or CD8+ T cells (Figure 1d, *P*=0.011 and *P*=0.014, respectively, and Figure 1e, *P*=0.041 and *P*=0.006, respectively). Besides the percentages of producing cells, the analysis of the MFI showed that Mo/Ma cultured with TIS and control-T cell increased NO and ROS production relative to Mo/Ma cultured alone (Figures 1d and e). Altogether these results indicate that both TIS and control-T cells promote ROS and NO production by Mo/Ma, but TIS-T cells have a stronger effect.

### TIS-T lymphocytes enhance the pro-inflammatory response of Mo/Ma

**Figure 2:**
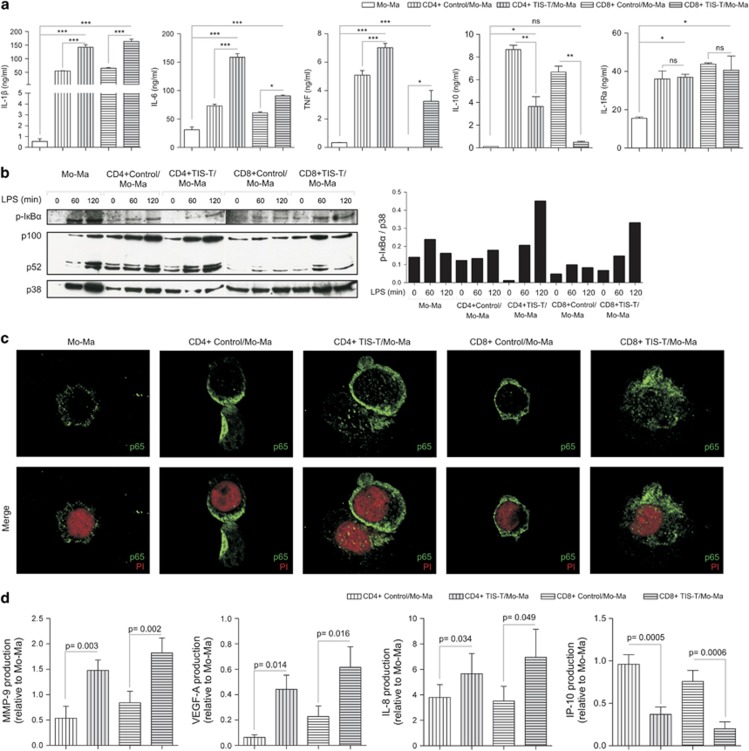
CD4+ and CD8+ TIS-T cells promote pro-inflammatory cytokines and angiogenic factors production by monocytes/macrophages. Monocytes were cultured as described in Figure 1. (**a**) After 40 h of co-culture, LPS was added and 48 h later cytokine production was measured. Graphics show pro-inflammatory cytokines (IL-1*β*, IL-6 and TNF) and anti-inflammatory cytokines (IL-10 and IL-1Ra) production. All results are shown as mean±S.E.M. *P<0.05, **P<0.01, ***P<0.001, ns, not significant (*P*>0.05); statistical analysis were performed using one-way ANOVA test. Data of seven experiments performed with different donors are shown. (**b**) After 40 h, co-cultures were either left as is or LPS stimulated for 60 and 120 min. Afterward, co-cultures were depleted of T cells, and total Mo/Ma lysates were separated on SDS–PAGE gels and immunoblotted with antibodies against the indicated proteins. Anti-p38 antibodies were used for equal loading control. The densitometric protein levels of phospho-I*к*B*α* were normalized to the respective levels of p38 and indicated in the bar graph. (**c**) After 40 h, co-cultures were LPS stimulated for 120 min. Then, T cells were depleted and Mo/Ma was stained as indicated. Confocal microscopy photographs ( × 60) show p65 protein (green) and nuclear (propidium iodide) (red) staining. Data are representative from three independent experiments. (**d**) After 40 h of co-culture, LPS was added and 48 h later, the production of angiogenic (MMP-9, VEGF-A and IL-8) and angiostatic (IP-10) factors was measured. Graphics show the average of angiogenic or angiostatic factor production in culture supernatant of Mo/Ma co-incubated with TIS-T cells or control T cells relative to culture supernatant of Mo/Ma cultured alone (*n*=7). Statistical analyses were performed using one-tailed paired *t-*test. *P-*values (TIS-T/Mo-Ma. co-culture *versus* control T/Mo-Ma co-culture) are indicated on graphics

Inflammatory responses have decisive roles at different stages of tumor development, including initiation, promotion, malignant conversion, invasion and metastasis. Most, if not all, solid malignancies promote inflammation through the secretion of molecules, which activate macrophages through toll-like receptor 4 (TLR4) and TLR2.^17^ Thus, considering that an inflammatory microenvironment is a hallmark component of all tumors, we sought to determine whether CD4+ or CD8+ TIS-T lymphocytes can modulate the effector function of Mo/Ma triggered by a powerful pro-inflammatory stimulus such as LPS. Compared with those cultured alone or with control-T lymphocytes, LPS-stimulated Mo/Ma co-cultured with CD4+ or CD8+ TIS-T cells produced increased amounts of pro-inflammatory cytokines such as IL-1*β*, IL-6 and TNF and decreased amounts of the anti-inflammatory cytokine IL-10. In contrast, both TIS- and control-T cells similarly promoted IL-1Ra secretion by Mo/Ma (Figure 2a). To exclude TIS-T cells as a potential source of cytokines in the CC assays, we evaluated the identity of the cytokine producing cells by flow cytometry. Remarkably, >90% of cells within the TNF-, IL-6- or IL-1*β*-producing population detected in the TIS-T cells/Mo-Ma CCs were CD14+, while CD3+ cells were only found in non-cytokine producing population (Supplementary Figure 1a).

The enhanced pro-inflammatory cytokine response to LPS observed in CD4+ or CD8+ TIS-T cells-modulated Mo/Ma could not be explained by an increased CD14 nor TLR-4 expression (Supplementary Figures 2a and b) but correlated with a greater activation of the canonical pathway of NF-*κ*B. Indeed, upon LPS stimulation, Mo/Ma modulated by CD4+ or CD8+ TIS-T lymphocytes showed an increased expression of phospho-I*κ*B*α* with respect to those co-incubated CD4+ or CD8+ control-T lymphocytes, which became even more evident after 120 min of stimulation (Figure 2b). In concordance, we detected that TIS-T-modulated Mo/Ma exhibited higher p65 translocation (Figure 2c). In the same line of evidence we observed that, upon LPS stimulation, Mo/Ma co-incubated with CD4+ and CD8+ TIS-T lymphocytes exhibited higher intracellular tyrosine phosphorylation (Supplementary Figure 2c). The activation of the non-canonical pathway of NF-*κ*B was evaluated by the expression of p100 and p52 and no differences were detected between Mo/Ma co-incubated with TIS- or control-T lymphocytes (Figure 2b).

### TIS-T lymphocytes promote the production of angiogenic factors and inhibit IP-10 secretion by Mo/Ma

Considering that the activation of the canonical pathway of NF-*κ*B has been implicated in the induction of pro-tumorigenic mediators,^18^ we determined the production of angiogenic factors such as MMP-9, VEGF-A and IL-8 by LPS-stimulated Mo/Ma co-cultured with TIS-T lymphocytes. Figure 2d shows that the normalized amounts of MMP-9 quantified in the culture supernatants of LPS-stimulated CD4+ or CD8+ TIS-T cell/Mo-Ma CCs (relative to Mo/Ma cultured alone) were significantly higher than those detected in supernatants of CD4+ or CD8+ control-T cell/Mo-Ma CCs. Similar to MMP-9, the amounts of VEGF-A and IL-8/CXCL8 measured in culture supernatant of LPS-stimulated CD4+ or CD8+ TIS-T cell/Mo-Ma CCs were significantly higher than those quantified in the culture supernatant of LPS-stimulated control-T cells/Mo-Ma CCs. As we previously did, we excluded T cells as possible sources of MMP-9 and IL-8 in the TIS-T lymphocyte/Mo-Ma CCs by determining, after intracellular staining, that >90% of the MMP-9 or IL-8 producing cells were CD14+ (Supplementary Figure 1b). In addition, we observed that anti-CD3/anti-CD28-stimulated CD4+ or CD8+ TIS-T cells do not produce VEGF-A (data not shown).

We next evaluated the production of IP-10/CXCL10, a factor commonly produced by monocytes that exhibits a potent angiostatic activity and inhibits tumorigenesis.^19^ In the culture supernatant of Mo/Ma co-cultured with CD4+ and CD8+ TIS-T lymphocytes we detected a reduced concentration of IP-10 with respect to culture supernatants of Mo/Ma cultured with control-T cells (Figure 2d).

**Figure 3:**
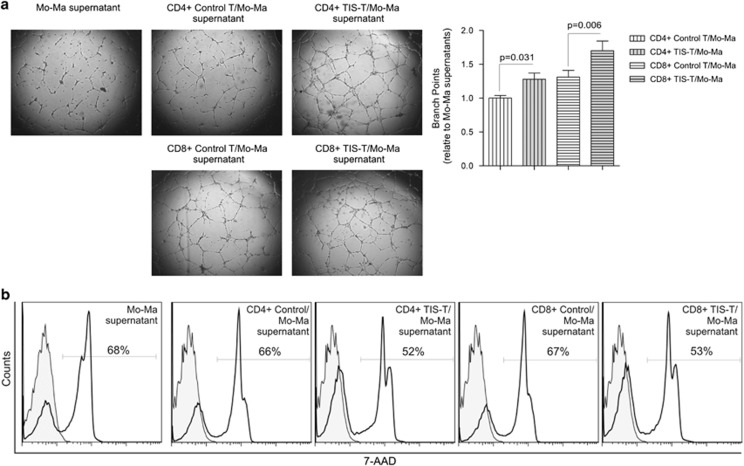
Culture supernatants of TIS-T-modulated monocytes/macrophages promote pro-angiogenic responses. (**a**) HMEC-1 were seeded on plates coated with Matrigel and cultured in EGM-2 supplemented with supernatants of TIS-T or control T cell/Mo-Ma co-cultures or supernatants of Mo/Ma cultured alone. After 18 h, tube formation was examined and the total number of branch points was quantified on the entire surface of each well. Bar graph shows the number of branch points elicited by control T or TIS-T/Mo-Ma co-culture supernatants relative to culture supernatants of Mo/Ma cultured alone (*n=*3). Statistical analyses were performed using one-tailed paired *t-*test. *P-*values (TIS-T/Mo-Ma co-cultures *versus* control T/Mo-Ma co-cultures) are indicated on graph. (**b**) HeLa cells were treated with mitomycin C to inhibit cell proliferation. Then, HeLa cells were cultured with supernatant of TIS-T or control T cell/Mo-Ma co-cultures or supernatants of Mo/Ma cultured alone. After 48 h HeLa cell death was measured by flow cytometry by using 7-AAD labeling. In all cases, fill histograms represent unstained HeLa cells. Data are representative from three independent experiments

We next evaluated the pro-angiogenic effects of the supernatants from TIS-T cells/Mo-Ma CCs in assays of tubulogenesis and wound healing. The capillary-tube formation elicited by the culture supernatants from CD4+ or CD8+ TIS-T-modulated Mo/Ma was significantly augmented with respect to that induced by supernatant from control cultures (Figure 3a). In addition, we observed that wound healing of Human microvascular endothelial cells (HMEC) treated with supernatants from CD4+ or CD8+ TIS-T cell/Mo-Ma CCs exhibited a similar wound closure than HMEC treated with supernatants of control-T cells/Mo-Ma CCs or Mo/Ma cultured alone (data not shown).

To establish whether factors present in supernatant of TIS-T cell/Mo-Ma CCs may have any impact on tumor cell death. We evaluated cell death of HeLa cells treated with different culture supernatants. We observed that after 48 h of culture, HeLa cells incubated with supernatant of Mo/Ma co-cultured with CD4+ or CD8+ TIS-T cells showed a reduced cell death percentage compared with the corresponding controls (Figure 3b). Altogether these results indicate that some mediator/s present in the culture supernatant of TIS-T cell/Mo-Ma CCs promote tubulogenesis and survival of tumor cells but not wound healing. These findings are not conflictive as the molecular mechanisms involved in tubulogenesis and wound healing are not identical.^20^

### TIS-T lymphocytes modulate Mo/Ma through Tim-3/galectin-9 (Gal-9) and CD40L/CD40 pathways

**Figure 4:**
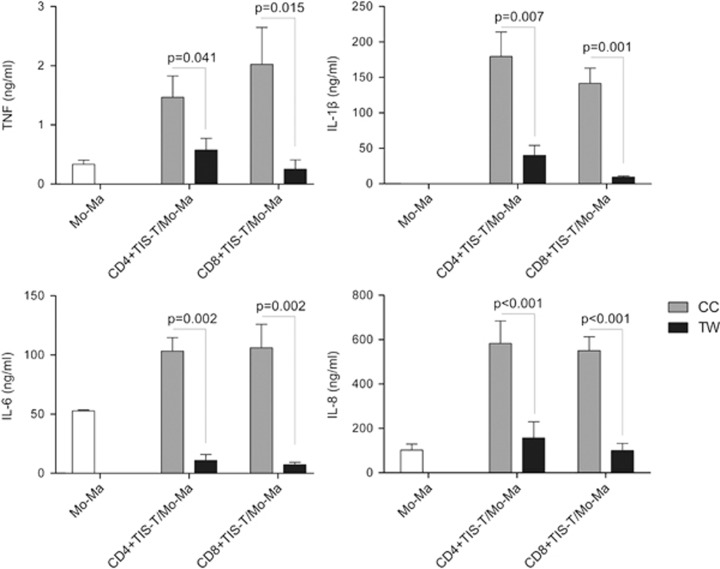
Monocytes/macrophages modulation by CD4+ and CD8+ TIS-T cells requires cell-o-cell contact. Monocytes were co-cultured with CD4+ or CD8+ TIS-T cells in the same well (CoCulture, CC, gray bars) or cultured separately by a Transwell (TW, black bars), with stimulated CD4+ or CD8+ TIS-T cells in the insert and monocytes in the lower well. After 40 h of co-culture, LPS was added and 48 h later TNF, IL-1*β*, IL-6 and IL-8 were measured. Bars represent average of cytokine/angiogenic factor production±S.E.M. (*n*=3). Statistical analyses were performed using one-tailed paired *t-*test. *P-*values (CC *versus* TW) are indicated on each graph

To investigate whether Mo/Ma modulation mediated by TIS-T lymphocytes requires signals derived from cell contact or soluble factors, we performed TIS-T cell/Mo-Ma CCs as before and also in a transwell system (TW). The disruption of physical contact between monocytes and CD4+ or CD8+ TIS-T cells completely abrogated the ability of TIS-T cells to potentiate Mo/Ma production of TNF, IL-1*β*, IL-6 and IL-8 (Figure 4).

**Figure 5:**
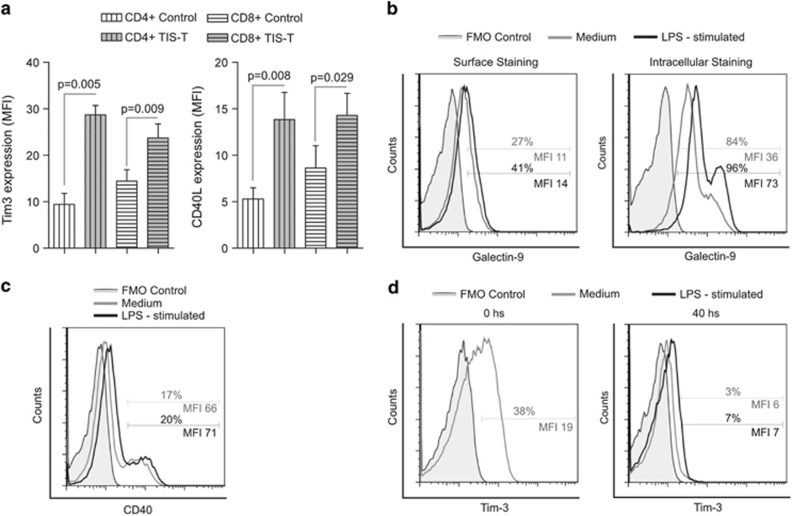
Phenotypic profiling of CD4+ and CD8+ TIS-T cells and monocytes/macrophages. (**a**) Tim-3 and CD40L expression on stimulated control T cells and TIS-T cells. Bars represent Tim-3 or CD40L expression (MFI±S.E.M.) (*n=*6). Statistical analyses were performed using one-tailed paired *t-*test. *P-*values (TIS-T cells *versus* control T cells) are indicated on graph. (**b**–**d**) Monocytes were culture 40 h and then were either unstimulated or LPS-stimulated for 18 hours. (**b**) Representative histograms of Gal-9 expression on surface and intracellulary on LPS-stimulated or non-stimulated monocytes. (**c**) Representative histogram of CD40 expression on LPS-stimulated or non-stimulated monocytes. (**d**) Representative histograms of Tim-3 expression on recently isolated human monocytes (0 h, before culture) and on LPS-stimulated or non-stimulated monocytes. In all cases, fill histograms represent FMO control. Representative histograms are shown from one donor out of at least three different donors

To further elucidate the pathways implicated in Mo/Ma modulation by TIS-T lymphocytes, we performed a phenotypic profiling of CD4+ and CD8+ TIS-T cells and Mo/Ma focusing on surface molecules involved in the pro-inflammatory cytokine induction in Mo/Ma.^21, 22, 23^ We observed that after anti-CD3 stimulation, both CD4+ and CD8+ TIS-T cells exhibited a higher expression of Tim-3 and CD40L than stimulated CD4+ and CD8+ control-T cells (Figure 5a). Remarkably, we determined that Mo/Ma stimulated with LPS under our *in vitro* conditions expressed, on the cell surface as well as intracellulary, Gal-9, the known ligand of Tim-3^24^ (Figure 5b). Furthermore, 20% of the LPS-stimulated CD14+ population expressed CD40, the receptor of CD40L (Figure 5c).

In addition, we confirmed previous observations by Zhang *et al.*^25^ that showed that CD14+ Mo/Ma exhibited a fairly high level of Tim-3 expression; however, it is significantly downregulated in the CC conditions (Figure 5d).

**Figure 6:**
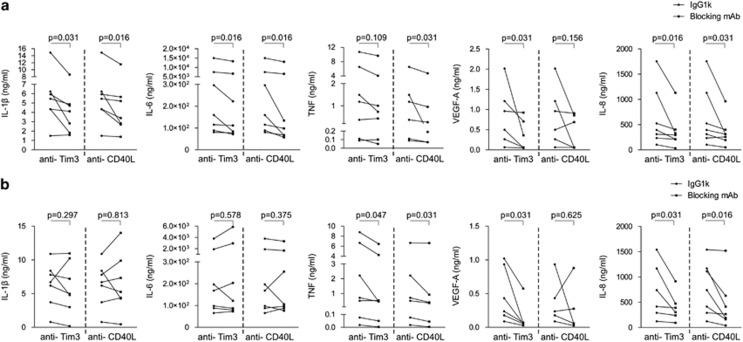
TIS-T cells modulate monocytes/macrophages through Tim-3/Gal-9 and CD40L/CD40 pathways. Monocytes were cultured with CD4+ or CD8+ TIS-T cells in the presence of blocking mAb against Tim-3 or CD40L molecules or IgG1*κ* isotype control. After 40 h LPS was added and 48 h later cytokines and angiogenic factors were measured. Cytokine and angiogenic factors production in CD4+ TIS-T/Mo-Ma (**a**) and CD8+ TIS-T/Mo-Ma (**b**) co-cultures, in the presence of isotype control paired to blocking mAb. Statistical analysis was performed by using Wilcoxon signed rank test. *P-*values (TIS-T/Mo-Ma co-cultures with IgG1*κ* isotype control *versus* TIS-T/Mo-Ma co-cultures with blocking mAb) are indicated on graphs

To test whether Tim-3/Gal-9 and CD40L/CD40 pathways participated in the interaction among TIS-T lymphocytes and Mo/Ma that favored production of pro-inflammatory cytokines and angiogenic factors, monocytes and TIS-T lymphocytes were cultured in the presence of blocking antibodies against Tim-3 or CD40L. We observed that IL-1*β*, IL-6, VEGF-A and IL-8 production by Mo/Ma co-cultured with CD4+ TIS-T was significantly reduced after anti-Tim-3 treatment (median reduction: 12%, 11%, 59% and 36%, respectively) (Figure 6a). Furthermore, CCs with cells from five out of seven donors reduced TNF secretion upon anti-Tim-3 treatment. However, inclusion of the non-responding donors in the statistical analysis ended in a non-significant difference. Blockade of the CD40L/CD40 interaction resulted in a reduced IL-1*β*, IL-6, TNF and IL-8 production in CD4+TIS-T cell/Mo-Ma CCs (median reduction: 22%, 17%, 37% and 38%, respectively). Again, most of the CCs from different donors (five out of six) showed reduced amounts of VEGF-A after anti-CD40L treatment (median reduction: 31%), though the difference considering all donors was not statistically significant.

Mo/Ma co-cultured with CD8+ TIS-T lymphocytes showed a slightly different response to the presence of blocking mAbs than those co-cultured with CD4+ TIS-T cells. Thereby, we observed that anti-Tim3 mAb was able to significantly reduce the secretion of TNF, VEGF-A and IL-8 (median reduction: 36, 70 and 23%, respectively) but not of IL-1*β* and IL-6, although most of the CCs from different donors showed a trend toward a reduced production of these cytokines. Moreover, anti-CD40L treatment significantly reduced the amounts of TNF and IL-8 in these CCs (median reduction: 41% and 61%, respectively), whereas it had no evident effect on IL-1*β*, IL-6 or VEGF-A production (Figure 6b).

## Discussion

Previously it has been demonstrated that TAMs are a heterogeneous cell population that acquire different phenotypes influenced by cancer cells, surrounding stroma as well as immune system cells which infiltrate tumors.^7^ Interactions between TAMs with tumor infiltrating-Tregs, B cells and Th2 cells have been previously described.^1^ However, the interaction between senescent T cells and macrophages and their biological relevance has not been studied so far. Our work provides evidence for previously uncharacterized roles of human CD4+ and CD8+ TIS-T lymphocytes in the modulation of macrophage activity. Thus, TIS-T lymphocyte emerges as a new relevant player in the complex interaction between tumor and tumor-infiltrating cells that influences tumor progression.

The functional diversity of macrophages is critical to orchestrate the variety of effector functions that range from inflammation and phagocytosis to immunoregulation and tissue remodeling. Particularly in the tumor microenvironment, the phenotype and function of TAMs have important consequences for tumor progression.^26^ Indeed, M1 macrophages are efficient immune cells able to kill tumor cells and to promote anti-tumor responses.^7^ However, M1 phenotype has also been associated with cancer initiation and promotion.^27^ Once the tumor is initiated and as it progresses toward malignancy, the macrophage phenotype has been shown to change to the M2 phenotype. Accordingly, it has been demonstrated that TAMs are composed of distinct populations that share features of M1 and M2 types.^28, 29^

It has been demonstrated that T lymphocytes modulate macrophage function *in vivo* and *in vitro*. Thus, by producing IFNγ, Th1 cells can drive classical M1 polarization, while Th2 cells direct M2 polarization through IL-4, IL-13 and IL-33.^7, 30^ Studies performed by Tiemessen *et al.*^9^ highlighted additional T cell subsets able to modulate Mo/Ma function. These authors demonstrated that human monocytes cultured in the presence of Tregs cells differentiate into M2-like macrophages. Similarly, we observed that after co-incubation with CD4+ or CD8+ TIS-T lymphocytes, Mo/Ma acquire some effector response commonly attributed to the M2 phenotype. These attributes include high production of angiogenic factors such as IL-8, VEGF-A and MMP-9 together with low secretion of IP-10. However, TIS-T lymphocytes promoted an uniform increment of CD16 expression on the CD14+ Mo/Ma, and co-expression of CD14 and CD16 is used to define a sub-population of pro-inflammatory monocytes.^31^ This finding, together with augmented production of NO, ROS and pro-inflammatory cytokines by TIS-T cell-modulated Mo/Ma, indicated that CD4+ or CD8+ TIS-T cells may also imprint monocytes with attributes of inflammatory M1. Altogether, our results demonstrate that the interaction between TIS-T cells and monocytes results in the differentiation of a Mo/Ma population with mixed M1 and M2 features.

Numerous reports link inflammation with tumorigenesis, angiogenesis and metastasis.^10^ Based on that, it could be speculated that, within the tumor microenvironment, TIS-T cell/macrophage interaction may certainly have important consequences for tumor progression since TIS-T cells promote the production of mediators such as TNF, IL-1*β* and IL-6. Particularly, TNF is a key cytokine involved in inflammation, immunity, cellular homeostasis and tumor progression.^32^ Regarding IL-6 and IL-1*β*, they are pleiotropic cytokines with a broad range of functions that have been shown to contribute to a microenvironment that promotes angiogenesis and inflammation in cancer.^10^ The pro-angiogenic function of inflammatory cytokines such as TNF and IL-1*β* is mediated by the upregulation of angiogenic factors such as VEGF, IL-8 and MMPs in vascular endothelial, tumor and inflammatory cells.^33^ Indeed, considerable evidence indicates that TAMs have an important role in regulating angiogenesis within the tumor microenvironment as they release a number of potent pro-angiogenic cytokines and growth factors such as VEGF, TNF, IL-8 and bFGF, as well as MMPs.^34, 35, 36^ In addition to inflammatory cytokines, Mo/Ma modulated by TIS-T lymphocytes exhibit an increased production of VEGF-A, IL-8 and MMP-9. Furthermore, we observed that culture supernatants from Mo/Ma co-incubated with CD4+ or CD8+ TIS-T lymphocytes have pro-angiogenic effects as determined by their increased tubulogenesis and tumor cell pro-survival potential. In spite of not identifying the mediator/s, our results are in agreement with the findings that pro-inflammatory cytokines and angiogenic factors provide anti-apoptotic signals that prevent tumor cell death.^37, 38, 39^

Mo/Ma modulation mediated by TIS-T lymphocytes requires cell-to-cell contact. Although we cannot rule out the involvement of additional pathways, we specifically demonstrated that Tim-3/Gal-9 and CD40L/CD40 interactions participate in this process. Interestingly, our results indicate that CD4+ and CD8+ TIS-T lymphocytes use different molecules to interact with Mo/Ma and to regulate particular aspects such as secretion of pro-inflammatory cytokines and angiogenic factors. The consequences of Tim-3 signaling on T cells have been the focus of numerous studies;^40^ however, recent reports established that there is a reciprocal signal transmitted to the participating Gal-9-expressing antigen presenting cell. Jayaraman *et al.*^22^ demonstrated that Tim-3 expressed on Th1 cells interacts with its ligand Gal-9 expressed by *Mycobacterium tuberculosis*-infected macrophages to restrict intracellular bacterial growth. Using an *in vitro* assay and *in vivo* model of *M. tuberculosis* infection, these authors demonstrated that Tim-3/Gal-9 interaction stimulates a significant production of IL-1*β*, IL-6, TNF, MIP1*αβ* and G-CSF by infected macrophages. In addition, several studies reported the impact of CD40L/CD40 interaction on macrophage response. Engagement of CD40 on M1 macrophages induces the expression of NO and TNF.^41, 42^ In this context, demonstrating the participation of Tim-3/Gal-9 and CD40L/CD40 pathways on the monocyte/macrophage modulation induced by TIS-T cells may contribute to decipher the role of macrophages in human tumors and may have therapeutic implications.

Treatment with anti-Tim-3 mAbs has been tested *in vitro* in patients with melanoma and hepatocellular carcinoma as a strategy to overcome T cell exhaustion and potentiate anti-tumor immune response.^3^ In light of our results, we suggest that the inhibition of Tim-3/Gal-9 pathway may affect the effector function of different leukocyte subsets. Unraveling the effects of anti-Tim-3 treatment on these several subsets will be important for predicting the outcome of this approach in cancer immunotherapy. Activation of CD40 pathways on macrophages may have a dual role in the survival and invasive capacity of tumor cells.^43^ However, several reports indicate that CD40-activated macrophages produce inflammatory mediators that promote chronic inflammation.^44^ In the same direction, we observed that the CD40L/CD40 pathway is involved in the enhancement of inflammatory activity of macrophages induced by TIS-T cells.

In summary, our results provide evidence for a previously uncharacterized role of TIS-T cells in human Mo/Ma modulation that may impact on tumor progression. Considering that macrophages are critical mediators of cancer-related inflammation, focusing on this relationship between TIS-T cells and macrophages may alter the balance of the type of tumor inflammation and create opportunities for better anti-tumor responses. Thus, future studies should be directed to better understand macrophage functional changes induced by TIS-T lymphocytes to best tailor the immune response against cancer.

## Materials and Methods

### Cell culture

Human peripheral blood mononuclear cells (PBMCs) from healthy donors (age 25–40) were isolated by centrifugation over Ficoll-Hypaque gradients (GE Healthcare Bio-Science AB, Uppsala, Sweden). CD4+ T cells, CD8+ T cells and monocytes were purified by positive selection from total PBMCs using CD4, CD8 or CD14 Microbeads (Miltenyi Biotech, San Diego, CA, USA) following manufacturer's instructions. The purity of each population was routinely checked and was >95% as determined by flow cytometry. Ethical approval was obtained from the Hospital Nacional de Clinicas Ethics Committee (16 October 2008). Purified CD4+ and CD8+ T cells and monocytes were cultured with RPMI-1640 media supplemented with 10% fetal bovine serum (Hyclone, Tauranga, New Zealand), 1% L-Glutamine (GlutaMax, Invitrogen Inc., Carlsbad, CA, USA) and 25 mmol/l Hepes (Cell Gro, Mediatech, Herndon, VA, USA). Cell viability was routinely checked using a dye exclusion test while counting cells.

The human solid tumor cell line Tu167 and human HeLa cell lines were cultured in RPMI-1640 and DMEM complete media, respectively, up to 90% confluence. HMEC-1 were grown in endothelial growth medium-2 (EGM-2, Lonza, Walkersville, MD, USA).

### Induction of senescence by tumor cell line

TIS-T cells were obtained as previously described.^15^ Briefly, CD4+ or CD8+ T lymphocytes were co-cultured for 6 h with Tu167 cells at a 1 : 1 Tumor/T cell ratio. Then, the T cells were collected, washed and cultured for 7 days in complete media. T cells collected after co-incubation with tumor cells were found to be 99% CD3+ by flow cytometry. Purified CD4+ or CD8+ T cells cultured for 7 days in complete media without a previous CC with the tumor cell line were used as controls.

### CCs and TW and blocking experiments

Monocytes and CD4+ or CD8+ TIS-T cells were co-cultured (1 : 1 ratio) in RPMI complete media for 40 h in the presence of anti-CD3 mAb (TR66, in house preparation). As controls, monocytes were cultured alone or co-cultured with CD4+ or CD8+ control-T cells (1 : 1 ratio). After 40 h, cultures were stimulated or not with LPS (50 ng/ml, InvivoGen *E. coli* 0111:B4) for an extra 48 h. TW experiments were performed culturing monocytes in the lower chamber and T cells were stimulated with anti-CD3/CD28/CD2 beads in the inserts (0.4 *μ*m pore size, Corning Life Sciences, Manassas, VA, USA). After the initial 40 h, the cultures were stimulated with LPS as described. For blocking experiments, monoclonal antibodies against Tim-3 (10 *μ*g/ml, F38-2E2, Biolegend, San Diego, CA, USA) and CD154 (10 *μ*g/ml, 24–31, Biolegend) or isotype controls (10 *μ*g/ml, MOPC-21, Biolegend) were added at the beginning of the CC.

### Flow cytometry

Mo/Ma were harvested and stained with anti-human CD14 AlexaFluor-647 or FITC, CD206 APC, Gal-9 PE, CD16 PE, CD40 PE-Cy5 (Biolegend), HLA-DR PE-Cy7, B7.H4 Biotin (eBioscience, San Diego, CA, USA) or isotype controls in PBS with 2% FSB on ice for 20 min. T cells were harvested and stained with mAb to human CD3 PerCP, CD27 APC-Alexa750, CD28 APC and Tim-3 PerCP-eFluor710 (eBioscience).

To detect CD40L expression, PE-labeled anti-human CD40L/CD154 antibodies were cultured with T cells in the presence of anti-CD3 mAb and Monensin solution (eBioscience) during 40 h. Intracellular staining for phosphotyrosine was performed an AlexaFluor-647-conjugated anti-human phosphotyrosine (PY20, Biolegend) antibody.

The production of ROS and NO was evaluated using the molecular probes: H_2_DCF-DA (10 *μ*M, Invitrogen Inc.) and DAF-FM DA (10 *μ*M, Molecular Probes, Inc.), respectively.

The assessment of phagocytosis was performed using 1 *μ*m-Fluoresbrite Yellow Green (YG) Carboxilate Microspheres (Polysciences, Inc., Warrington, PA, USA; 1 : 25 cell/microspheres ratio), which were added to CCs for 30 min. Afterwards, the uptake of YG-microspheres was evaluated in CD14+ cells.

All samples were acquired on a FACS Canto II (BD Biosciences, San Jose, CA, USA) and then analyzed with Flow Jo software (LLC, Ashland, OR, USA).

### Quantification of cytokines, angiogenic and angiostatic factors

Cytokines, angiogenic and angiostatic factors were measured in supernatant of LPS-stimulated Mo/Ma co-cultured with CD4+ or CD8+ TIS-T or control-T cells. TNF, IL-1*β*, IL-6 and IL-8 were measured by ELISA (Biolegend). VEGF-A, MMP-9 and IP-10 were measured by Flow Cytometry with Flow Cytomix Simplex kits (eBioscience).

### Western blot analysis

Monocytes and TIS-T and control-T cells were co-cultured for 40 h as previously described, and then cultured with medium or LPS for 60 and 120 min. Then, using a protocol widely described by Wahl *et al.*,^45^ that takes advantage of the ability of monocytes to adhere to plastic plates, T cells were removed by repeatedly washing with warm PBS 1 × . Then, adherent Mo/Ma (average purity 90%, routinely checked by flow cytometry) were lysed in 1 × SDS reducing sample buffer. Extracts were sonicated for 15 s and immediately separated on 10% SDS-PAGE gels, transferred to nitrocellulose membranes (BioRad Systems, Hercules, CA, USA) and incubated with anti-phospho-I*κ*B*α* (Ser32, 14D4), NF-*κ*B2 p100/p52 (Cell Signaling Technology, Danvers, MA, USA) or p38 mAbs (loading control, Sigma-Aldrich, St. Louis, MO, USA). Unstimulated Mo/Ma from CCs with control-T or TIS-T cells were immunoblotted with antibodies against TLR-4 (Abcam, Cambridge, MA, USA) and *β*-actin (Cell Signaling Technology). In all cases, protein bands were detected by enhanced chemiluminescence (Thermo Scientific Pierce, Rockford, IL, USA). The protein band intensities were analyzed using GelPro Analyzer software (Media Cybernetics, Warrendale, PA, USA) and normalized to the p38 or *β*-actin band.

### Tumor-cell survival

HeLa cells were treated with Mitomycin C (75 *μ*g/ml) for 30 min to inhibit cell proliferation. HeLa cells were cultured with supernatant of TIS-T or control-T cell/Mo-Ma CCs or supernatants of Mo/Ma cultured alone during 48 h. Then, we evaluated HeLa cell death/survival was then evaluated by flow cytometry staining with 7- aminoactinomycin D (7-AAD from BD Pharmingen, San Diego, CA, USA) during 15min.

### *In vitro* tube formation assay

HMEC-1 cells were seeded at a density of 1.5 × 10^4^ cells/well on plates coated with growth factor-reduced basement membrane matrix (Matrigel, BD Biosciences) and cultured with supernatant of TIS-T or control-T cell/Mo-Ma CCs or supernatants of Mo/Ma cultured alone. After 18 h, tube formation was examined by phase-contrast microscopy and the total number of branch points was quantified by using ImageJ software (NIH, Bethesda, MD, USA).

### Cytospin and immunofluorescence microscopy

After 40 h, TIS-T or control-T cell/Mo-Ma CCs were stimulated with LPS for 120 min, then T cells were removed and Mo/Ma were centrifuged onto positively charged slides by cytospin, fixed and permeabilized. After blockade, fixed cells were incubated with rabbit anti-p65 antibodies (eBiosciences), AlexaFluor488-conjugated anti-rabbit IgG antibody (Invitrogen Inc.) and propidium iodide (BD Biosciences) for nuclear staining. Finally, stained slides were mounted with FluorSave (Calbiochem, Boston, MA, USA) and analyzed by confocal microscopy (Olympus Fluoview 300, Olympus America Inc., Center Valley, PA, USA).

### Statistical analysis

Statistical analysis was performed with GraphPad Prism 5.0 software (San Diego, CA, USA) by using One-way ANOVA and parametric or non parametric paired *t*-test according to the data distribution. *P*-values<0.05 were considered significant.

## Abbreviations

CD40L: CD40 ligand
Gal-9: galectin-9
MFI: mean of fluorescence intensity
MHC: major histocompatibility complex
Mo/Ma: monocytes/macrophages
NO: nitric oxide
PBMCs: peripheral blood mononuclear cells
ROS: reactive oxygen species
TAM: tumor-associated macrophages
Tim-3: T-cell immunoglobulin and mucin protein 3
TIS-T cells: tumor-induced senescent T cells
TLR: toll-like receptor
Tregs: regulatory T cells
