# Supplementary figures and images for: Tumor-induced senescent T cells promote the secretion of pro-inflammatory cytokines and angiogenic factors by human monocytes/macrophages through a mechanism that involves Tim-3 and CD40L
Source: Cell Death Dis. 2014 Nov 6;5(11):e1507–. doi: 10.1038/cddis.2014.451 (PMC4260722; doi:10.1038/cddis.2014.451)

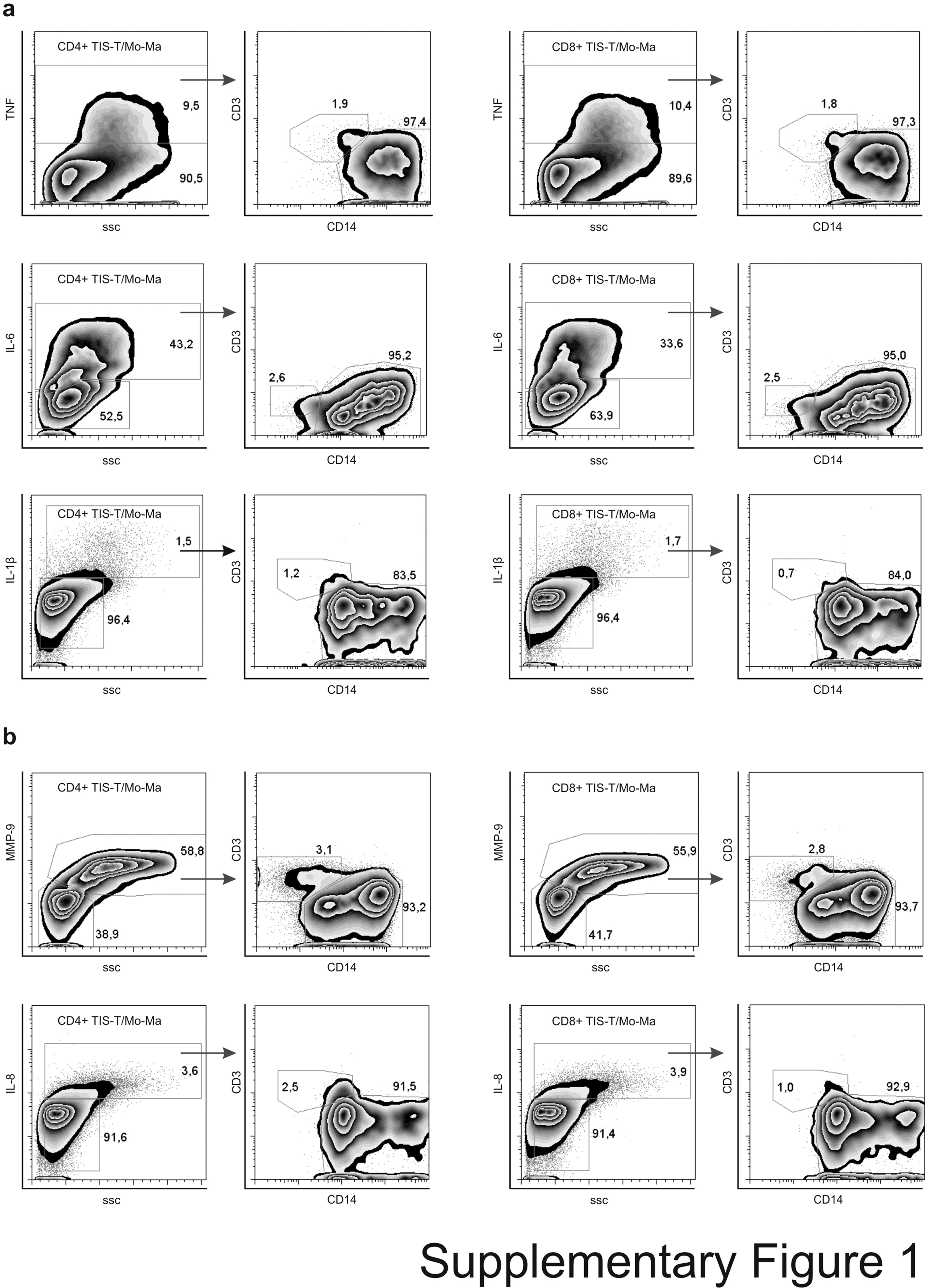

Supplement: Supplementary Figure 1 [file cddis2014451x1.tif]

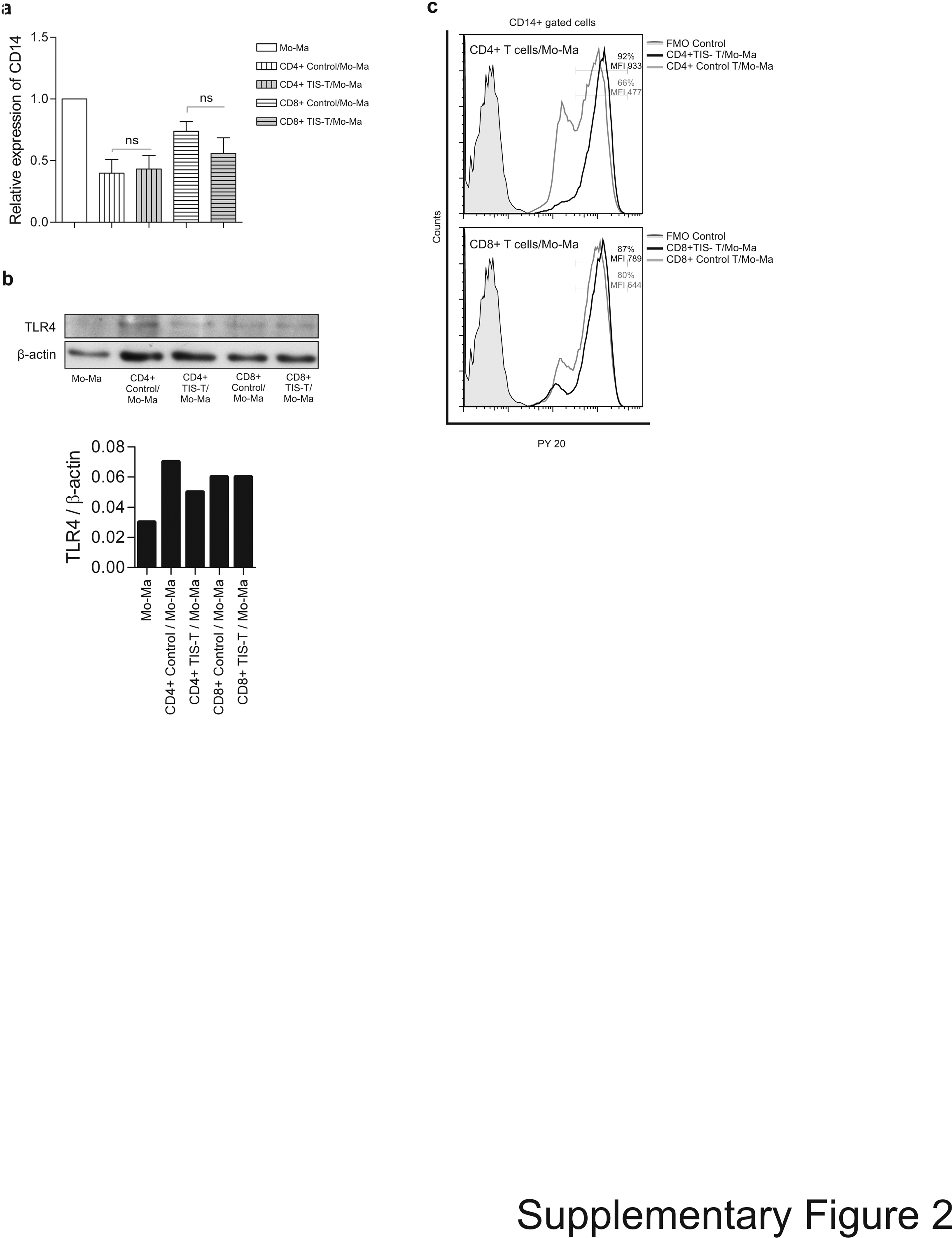

Supplement: Supplementary Figure 2 [file cddis2014451x2.tif]
