# Supplementary material for: Tumor-induced senescent T cells promote the secretion of pro-inflammatory cytokines and angiogenic factors by human monocytes/macrophages through a mechanism that involves Tim-3 and CD40L
Source: Cell Death Dis. 2014 Nov 6;5(11):e1507–. doi: 10.1038/cddis.2014.451 (PMC4260722; doi:10.1038/cddis.2014.451)
Supplement: Supplementary Figures [file cddis2014451x3.doc]

**Supplementary Figure 1**. **CD4+ and CD8+ TIS-T cells promote the production of pro-inflammatory cytokines and angiogenic factors by monocytes/macrophages**. Monocytes were cultured with CD4+ or CD8+ TIS-T cells (in 1:1 ratio), in the presence of anti-CD3 mAb (2μg/ml) for 40 hours. After that, co-cultures were stimulated with LPS for 5 hours in presence of Brefeldin A or Monensin for the last 3 hours. Then, cells were surface-stained with anti-CD3 and anti-CD14 antibodies, fixed and permeabilized with Citofix/Cytoperm and Perm Wash kit according to manufacturer’s instruction. Intracellular staining was performed using antibodies against cytokines (TNF, IL-6 and IL-1β) and angiogenic factors (MMP-9 and IL-8). Dot plots show intracellular cytokine expression (**a**) or intracellular angiogenic factor expression (**b**) on CD3+ and CD14+ populations. Representative dot plots of one out of 3 donors.

**Supplementary Figure 2**. **CD4+ and CD8+ TIS-T cells enhanced the pro-inflammatory response of monocytes/macrophages**. Monocytes were cultured as indicated in Supplementary Figure 1. After 40 hours, CD14 and TLR4 expression in monocytes were evaluated by Flow Cytometry and Western Blot, respectively. (**a**) CD14 expression (average MFI ± SEM) on Mo/Ma co-culture with TIS-T cells or Control T cells relative to Mo/Ma cultured alone. ns no significant (*p*>0.05); statistical analysis were performed by using One way ANOVA test (*n*=6). (**b**) After 40 hours T cells were depleted and total Mo/Ma lysates were separated on SDS-PAGE gels and immunoblotted with anti-TLR4. β-actin was used for equal loading control. The densitometric protein levels of TLR4 were normalized to the respective levels of β-actin and indicated in the bar graph. Results are representative of 3 independent experiments. (**c**) after 40 hours, co-cultures were stimulated with LPS for 60 minutes. Histograms represent intracellular tyrosine phosphorylation (PY20) on CD14+ gated cells. Results are representative of 3 independent experiments. In both cases, fill histograms represent Fluorescence Minus One (FMO) Control.
